# Generation of site-specific ubiquitinated histones through chemical ligation to probe the specificities of histone deubiquitinases

**DOI:** 10.3389/freae.2023.1238154

**Published:** 2023-07-31

**Authors:** Nouf Omar AlAfaleq, Yun-Seok Choi, Boyko S. Atanassov, Robert E. Cohen, Tingting Yao

**Affiliations:** ^1^ Department of Biochemistry and Molecular Biology, Colorado State University, Fort Collins, CO, United States; ^2^ Department of Pharmacology and Therapeutics, Roswell Park Comprehensive Cancer Center, Buffalo, NY, United States

**Keywords:** ubiquitin, histone, deubiquitinase, nucleosome, chemical ligation, ubiquitin-histone conjugate mimic, PTM, free ubiquitin sensor

## Abstract

The attachment of mono-ubiquitin to histones as a post-translational modification plays important roles in regulating chromatin structure and function. Like other epigenetic modifications, the site of ubiquitin attachment is critically important in determining its functional outcome. Depending on the type of histone and the specific lysine residue that is modified, ubiquitination acts in diverse pathways including DNA damage repair, transcription elongation, and transcription repression. Specific reader, writer and eraser activities have evolved to distinguish nucleosomes by ubiquitination of different sites. To facilitate biochemical studies of ubiquitinated nucleosomes, we have developed an efficient strategy to chemically ligate intact ubiquitin and histone proteins at specific sites to generate near-native ubiquitin-histone conjugates. Because these chemically-ligated ubiquitin conjugates are hydrolysable, they enabled us to characterize *in vitro* the specificities of several histone deubiquitinases. To gain insight into the mechanisms that contribute to the specificities of these deubiquitinases, we used a free Ub sensor-based real-time assay to determine their Michaelis-Menten kinetics. Our results confirmed previously reported specificities of BAP1 and USP22, but also revealed specificities of other histone deubiquitinases that have been less well defined in the literature.

## Introduction

The nucleosome, the basic unit of chromatin, regulates all processes that require access to DNA in eukaryotes. A nucleosome core particle (NCP) is comprised of approximately 147 base pairs of DNA wrapped in 1.67 turns around a histone octamer that contains two copies each of four core histones (H2A, H2B, H3 and H4) (Luger et al., 1997). A broad array of post-translational modifications (PTMs) have been discovered that attach to these histones and modify the properties of chromatin. One such modification is the addition of a single ubiquitin (Ub) protein to a lysine residue in the histones. Depending on the site of Ub attachment, these modifications have distinct functional consequences (Weake and Workman, 2008; Mattiroli and Penengo, 2021; Vaughan et al., 2021). Mono-ubiquitination at H2AK118 or K119 (amino acid positions are based on human histone sequences) is conserved in metazoans and plays a role in transcriptional repression and polycomb-dependent facultative chromatin. Ubiquitination at H3K14, K18 or K23 is implicated in the recruitment of DNA methyltransferase Dnmt1 and the maintenance of constitutive heterochromatin. In contrast, ubiquitination at H2BK120, a modification conserved throughout eukaryotes, is associated with actively-transcribed regions and has roles in transcription initiation, elongation and mRNA processing. A more recently discovered ubiquitination site at H2AK13 or K15 is conserved among vertebrates and is associated with the DNA damage response (DDR). In addition, other ubiquitination sites have been identified, and ubiquitination of all histone types has been observed (Vaughan et al., 2021).

Multiple deubiquitinases (DUBs) have been identified that potentially can reverse ubiquitination at different sites on histones. However, several factors have hampered efforts to understand the specificity and regulation of histone DUBs. One problem has been the difficulty to obtain homogenous ubiquitinated histones and nucleosomes for use as substrates *in vitro.* We and others have developed crosslinking-based approaches to install Ub at specific sites on histones (Chatterjee et al., 2010; Long et al., 2014; Morgan et al., 2019); although these Ub-histone analogs have facilitated many structural studies and advanced our understanding of how histone ubiquitination marks are “read” (Chen et al., 2022), the analogs’ non-hydrolyzable Ub-histone linkages preclude their use as DUB substrates. Strategies have been developed to produce native ubiquitinated histones that use a combination of expressed protein ligation (EPL) and solid phase peptide synthesis (SPPS) techniques (McGinty et al., 2008; Siman et al., 2013). Drawbacks, however, are that those protocols are technically challenging for most biochemistry laboratories and it is expensive to produce large quantities of proteins by SPPS. Recent efforts to characterize writers of histone ubiquitination have facilitated the production of enzymatically-active recombinant E3 ligases, but only for a few of the known histone ubiquitination forms (Uckelmann et al., 2018). Another problem is that many DUBs are regulated by associated factors *in vivo.* These accessory factors often are not present in the assays performed with recombinant enzymes produced in bacteria, thus undermining the biological relevance of *in vitro* assays. Mutations in multiple histone DUBs, such as BAP1, USP22, USP3, and USP16, have been linked to multiple forms of cancer (Aquila and Atanassov, 2020), making these DUBs attractive therapeutic targets. Reliable assays that reflect the biological activities of these enzymes are essential for development of meaningful high-throughput drug screens.

Here we describe an approach to obtain homogenous site-specific ubiquitinated H2A and H2B that were then reconstituted into nucleosomes and used to qualitatively and quantitatively characterize a panel of histone DUBs *in vitro*. Our results confirmed previously characterized specificities of BAP1 and USP22, but also revealed specificities of other histone DUBs that are less well defined in the literature; these findings underscore the importance of evaluating histone DUBs with chemically-defined substrates. We anticipate that our approach can be applied to generate other types of hydrolyzable Ub-histone conjugates regardless of the particular ubiquitination site or histone types, facilitating a variety of biochemical and structural studies of these important epigenetic marks, as well as development of therapeutic interventions targeting the enzymes that regulate them.

## Materials and methods

### Chemical reagents

N-(Allyloxycarbonyloxy)-succinimide (Alloc-OSu) was purchased from TCI Chemicals (Portland, OR). Chloro (pentamethylcyclopenta dienyl) (cyclooctadiene) ruthenium (II) ([Cp*Ru (cod)Cl]) was purchased from Sigma-Aldrich. N,N-diisopropylethylamine (DIEA) and N-hydroxysuccinimide was purchased from Acros Organics. Ethyleneimine was purchased from Chem Service (West Chester, PA). Thiophenol (99+%) was purchased from Alfa Aesar (Haverhill, MA).

### Cloning, expression and purification of HisUb

The pET3aHisUb plasmid was constructed to encode “HisUb”, human ubiquitin with 6xHis-Gly-Gly added to the N-terminus. BL21 (DE3)pLysS cells transformed with the pET3aHisUb plasmid were grown in 2X YT media supplemented with 100 μg/mL ampicillin and 34 μg/mL chloramphenicol at 37°C with shaking until an OD_600_ of 0.8–1 was reached. Expression of HisUb was induced by the addition of 0.4 mM IPTG, and cells were harvested after additional growth at 37°C for 4 h. Cell pellets were resuspended in lysis buffer (50 mM NaPi, pH 7.5, 500 mM NaCl, 10 mM imidazole, 1 mM PMSF, 1 mg/mL lysozyme, 15 μg/mL DNase I) and incubated on ice for 10–15 min. After sonication, the soluble fraction was separated from insoluble cellular debris by centrifugation at 12,000 *g* for 30 min at 4°C. 6xHis-affinity purification using Ni-NTA agarose (Qiagen) was performed according to the manufacturer’s instructions. The eluted HisUb was dialyzed against 10 mM Tris, pH 7.5, containing 0.2 mM EDTA and 50 mM NaCl, and further purified from minor contaminants by passing through 10 mL of Q Sepharose Fast Flow resin (GE Healthcare) pre-equilibrated in the dialysis buffer. HisUb was in the flow-through, which was further dialyzed against 20 mM NaPi, pH 7.5 and concentrated to 10 mg/mL before storing at −80°C. A yield of 200 mg typically was obtained from 6 L culture.

### Cloning, expression and purification of recombinant histones

All human histone mutants were generated by site-directed mutagenesis employing the QuikChange Site-Directed Mutagenesis kit (Agilent) per manufacturer’s protocol using histone plasmids reported previously (Long et al., 2014). All recombinant histones were expressed and purified according to Dyer et al. (Dyer et al., 2004). Those that contained cysteine were dialyzed into 1 mM acetic acid in the final step prior to lyophilization. Wild-type human H2A, H2B, H3.3, and H4 were purchased from the Histone Source at Colorado State University and used for reconstitution of histone dimers or octamers.

### Generation of site-specific ubiquitinated histones by chemical ligation

Below we describe the steps to generate H2BK_C_120Ub as shown in Figure 1. Similar procedures were used with H2AK119C or H2AK15C to generate ubiquitinated forms of H2A histones.

1) *Generation of HisUb-SR.* HisUb-SR was generated as described (El Oualid et al., 2010). Briefly, purified HisUb (**ɪ**, 4 mg, 1 mM) was incubated in 20 mM NaPi (pH 8.0) with 0.1 µM E1 Ub-activating enzyme, 10 mM ATP, 10 mM MgCl_2_, and 0.1 M fresh sodium 2-mercaptoethane-sulfonate (MESNA) at 37°C for 6 h. The E1 enzyme was then precipitated by the addition of 1/10 volume of glacial acetic acid, followed by centrifugation at 13,000 x *g* for 5 min. The supernatant fraction was then dialyzed at 4°C against 0.4% TFA/H_2_O followed by lyophilization.
2) *Alloc protection* was performed as described (Castaneda C. et al., 2011). Lyophilized HisUb-SR (**ɪɪ**, 4 mg) was dissolved in 0.4 mL anhydrous DMSO and incubated with 180 mM DIEA and 15 mM N-(Allyloxycarbonyloxy)succinimide (Alloc-OSu) in DMSO. The concentration of Alloc-OSu should equal the molar concentration of total amines. Each HisUb-SR molecule contains 1 N-terminal α-amino group, 7 histidines and 7 lysines, which give 15 possible sites for modification by Alloc. This reaction typically proceeded for 1 h at room temperature. Due to poor reactivity of histidine sidechains, the HisUb-SR derivatives obtained typically contained 10, 11, 12, 13, or 14 Alloc groups. The product mixture was then mixed with two volumes of ice-cold diethyl ether, vortexed for 15 s, the mixture allowed to settle, and the top organic layer was removed. This was repeated two more times to yield a white pellet. The pellet (**ɪɪɪ**) was dissolved in DMSO to 50 mg/mL (5.2 mM) for use in the subsequent chemical ligation reactions.
3) *Cysteine protection by MMTS in histones.* Lyophilized histone H2BK120C (**ɪv**, 10 mg) was dissolved in 2 mL 0.5% TFA/DMSO. MMTS (1 M stock in isopropanol, Thermo Scientific Pierce) was added to reach 3.5 mM (10-fold molar excess over the histone concentration) and DIEA was added to 75 mM to adjust the pH to ∼7. The solution was incubated at room temperature for 15 min. Complete protection of the cysteine to generate H2BK120C-S-S-CH3 (**v**) was confirmed by MALDI-TOF. The protein was then precipitated by diethyl ether as described for Alloc-protected HisUb-SR.
4) *Alloc protection of histones.* The pellet containing H2BK120C-S-S-CH3 (**v,** 10 mg) was dissolved in 2 mL DMSO and incubated with 240 mM DIEA and 161 mM Alloc-OSu. H2BK120C contains 19 lysines, 3 histidines and an N-terminal α-amino group, which give a total of 23 possible sites to be modified by Alloc. A 20-fold molar excess of Alloc-OSu was added to ensure complete protection, which typically was achieved in 2 h at room temperature. The resulting product, a mixture of H2BK120C-S-S-CH3 modified by 21, 22, and 23 Alloc groups, was precipitated by diethyl ether. The pellet (**vɪ**) was dissolved in 3.6 mL 7 M guanidine hydrochloride (GdnHCl) and 100 mM HEPES at pH 8.
5) *Cysteine deprotection and alkylation.* 20 mM TCEP was added to the dissolved histone (**vɪ**, 0.18 mM) and the solution incubated at room temperature for 15 min in order to deblock cysteines. The reduced cysteine was then alkylated by the addition of 55 mM ethyleneimine and incubated at 37°C for 1 h to generate Alloc-protected H2BK_C_120 (**vɪɪ**). The protein solution was diluted 3-fold with H_2_O, divided into 1.5 mg aliquots, and the protein precipitated with diethyl ether. The protein pellet was collected by centrifugation and washed with H_2_O twice to remove any remaining ethyleneimine. Each pellet (**vɪɪ**, 1.5 mg) was then dissolved in 75 µL 0.5% TFA/DMSO.
6) *Chemical ligation of HisUb and histone.* The ligation procedure was modified from (Castaneda C. et al., 2011). HisUb derivative **ɪɪɪ** (0.54 mg; 0.5 mM) was mixed with 1.5 mg of histone derivative **vɪɪ** (1 mM) in a total volume of 113 µL DMSO. This mixture was brought to 430 mM DIEA, 38 mM N-hydroxysuccinimide (from a fresh 1 M stock in DMSO), and 6 mM AgNO_3_ (from 0.1 M made fresh in DMSO). The reaction mixture was incubated at room temperature in the dark overnight followed by diethyl ether precipitation.
7) *Global Alloc deprotection.* The protein pellet from chemical ligation was dissolved in 0.3 mL 0.5%TFA/DMSO and the total concentration of Alloc moieties (from both histones and Ub) was calculated to be 10 mM; accordingly, 10% H2O and 10 mM [Cp*Ru (cod)Cl] (i.e., equivalent to total Alloc) and 10% v/v thiophenol were added and the mixture was incubated at 50°C for 4 h followed by the addition of 20% H_2_O and additional incubation for 3 h. Finally, the proteins were precipitated by ≥ 5 rounds of extraction with diethyl ether.

**FIGURE 1:**
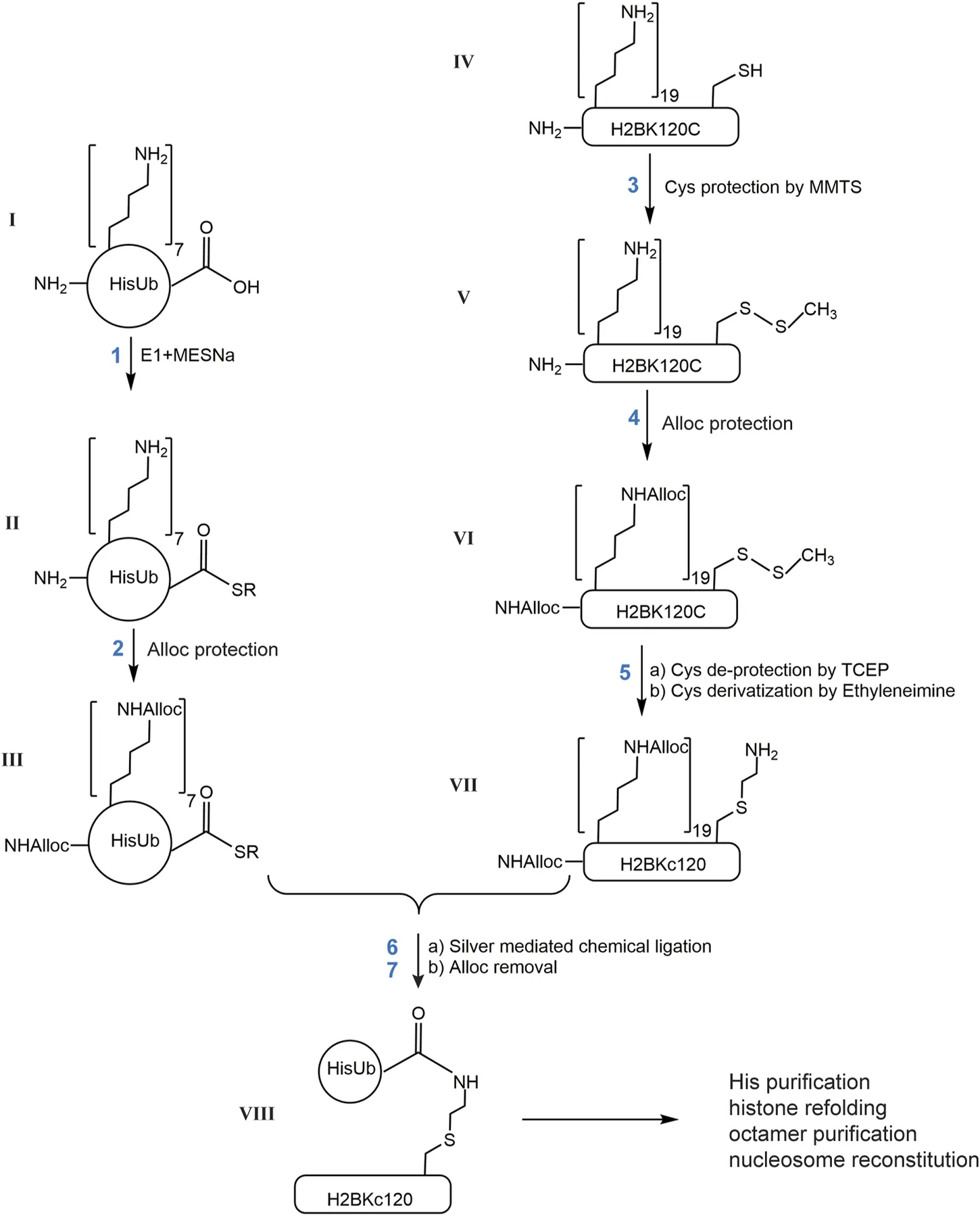
Scheme describing the generation of site-specific ubiquitinated histones by chemical ligation. Each reaction product is designated with a Roman number. R in II and III is ethylsulfonate.

### HisTrap purification of the Ub-histone product

The protein pellet after Alloc deprotection was dissolved in Buffer A (6 M GdnHCl, 5 mM β-mercaptoethanol, 0.1 M NaPi, pH 8, 10 mM imidazole) and loaded onto a 1 mL HisTrap column. After washing with 30 mL of Buffer A, the Ub-histone (**vɪɪɪ**) as well as unreacted HisUb were eluted by a gradient of 0–40% Buffer B (Buffer A+ 240 mM imidazole) over 20 mL. A 50 µL aliquot of each 0.5 mL fraction was analyzed by 15% SDS-PAGE after precipitation with methanol/chloroform (Wessel and Flugge, 1984) to remove GdnHCl prior to loading on the gel. Fractions containing the mixture of Ub-histone and HisUb were pooled and directly used in refolding with other core histones to form dimers or octamers. Free HisUb does not interfere with the refolding and was removed in subsequent gel filtration steps.

### Mass spectrometry

For HisUb or its derivatives, MALDI-TOF MS was performed on a Bruker Ultraflex MALDI-TOF/TOF mass spectrometer. Reaction intermediates were analyzed by ESI-MS on either of two instruments: 1) high resolution mass spectra of m/z 250–3,000 were acquired on a Thermo Scientific LTQ Orbitrap XL mass spectrometer using flow injection; 2) high resolution mass spectra of m/z 400–4,000 with resolution of 60,000 were obtained using a Thermo Scientific Orbitrap Velos mass spectrometer by direct infusion. Deconvolution of spectra used either the Xtract program in Xcalibur software or MagTran with the maximum charge set to 100.

Purified ubiquitinated histone dimers were analyzed by reverse-phase liquid chromatography coupled to a TOF LC/MS spectrometer. For HPLC fractionation, a Jupiter C4 column (Phenomenex) was used. The column was maintained at 40°C and initially equilibrated in 98% solvent A (0.05% TFA in water) and 2% solvent B (0.05% TFA in acetonitrile). The proteins were eluted at a flow rate of 100 μL/min using a program of 2 min at 2% B, followed by an 8-min linear gradient from 2 to 98% B for a total gradient time of 10 min. The effluent was directed to a 6224 TOF LC/MS spectrometer (Agilent Technologies). The data obtained were analyzed using the MassHunter Qualitative Analysis software B.05.00 (Agilent Technologies).

For MS/MS analysis of chemically ligated Ub-histone product, the Ub-histone band was cut from a Coomassie-stained 15% SDS-polyacrylamide gel and digested with trypsin following standard procedures. The resulting peptides were Zip-tip purified and concentrated. Subsequent chromatographic separation was performed on a reverse phase nanospray column (Thermo Scientific). The instrument was operated in orbitrap-LTQ mode where precursor measurements were acquired in the orbitrap (60,000 resolution) and MS/MS were acquired in the LTQ ion trap with ETD fragmentation. All MS/MS spectra were analyzed using Mascot (Matrix Science, London, UK; version 2.3.02). Mascot was set up to search a custom sequence database with additional modifications (i.e., amino alkylation of cysteine and amino alkylation + Gly-Gly). The results confirmed successful amino alkylation of cysteine to generate S-aminoethylcysteine and installation of Ub at the desired position.

### Refolding of histone dimers, octamers and nucleosome reconstitution

Refolding of histone dimers or octamers containing Ub-histone and subsequent nucleosome reconstitution followed procedures as described (Long et al., 2014). Histone octamers containing Ub-histones can be readily assembled with 147mer DNA containing the 601 Widom sequence (Lowary and Widom, 1998) to form nucleosomes by salt dilution (Owen-Hughes et al., 1999). Briefly, the 147mer DNA was prepared as described (Dyer et al., 2004). For each preparation, titrations were performed to determine the best DNA-to-histone octamer ratio for nucleosome formation. Typically, DNA and octamer were combined at 1:1 molar ratio in 10 μL refolding buffer to reach final concentrations of 1.5 μM DNA and octamer. Dilution buffer (10 mM Tris, pH 7.6, 1 mM EDTA, 1 mM DTT, and 0.1 mg/mL BSA) was added at 30 °C every 15 min with volumes of 3.3, 6.7, 5, 3.6, 4.7, 6.7, 10, 30 and 20 μL. The quality of the nucleosomes was evaluated by analyzing aliquots of the reactions by 6% native-PAGE (37.5:1 acrylamide:bis-acrylamide) run at 4 °C for 75 min at 130 V in 0.2X TBE. The gels were stained with ethidium bromide to visualize the nucleosomes and any free DNA.

### Cloning of 3XFlag-DUBs and generation of stable cell lines

The coding sequences of BAP1, USP3, USP16, and USP22 (Uniprot accession number Q92560, Q9Y6I4, Q9Y5T5, and Q9UPT9, respectively) were amplified by PCR using human cDNA as template with forward primers containing XhoI and reverse primers containing BamHI restriction sites.

*BAP1:* forward primer 5′-ATC​TAC​TCG​AGA​TGA​ATA​AGG​GCT​GGC​TGG A-3′ and reverse primer 5′- ACC​AAG​GAT​CCC​CCT​TAT​TCA​TTC​ACT​GGC​GCT​TG-3'

*USP3:* forward primer 5′- ATT​ATC​TCG​AGA​TGG​AGT​GTC​CAC​ACC​TGA​GC-3′ and reverse primer 5′- ACC​AAG​GAT​CCT​TAA​AGT​TTA​TCC​GAT​CCA​GCT​TTG​G -3'

*USP16:* forward primer 5′- ATT​ATC​TCG​AGA​TGG​GAA​AGA​AAC​GGA​CAA​AG -3′ and reverse primer 5′- ACC​ACG​GGA​TCC​TTA​CAG​TAT​TCT​CTC​ATA​AAA​TAG​GAG​G -3'

*USP22:* Forward primer 5′- AAC​AAC​TCG​AGA​TGG​TGT​CCC​GGC​CAG​A -3′ and reverse primer 5′- AAC​AAG​GAT​CCC​TAC​TCG​TAT​TCC​AGG​AAC​TGT​TTG​TGA -3'

The PCR products were cloned into a pcDNA5 FRT/TO vector containing an N-terminal 3XFlag tag sequence. Site-directed mutagenesis was used to introduce mutations at the catalytic site of each DUB: C91S in *BAP1*, C168S in *USP3*, C205S in *USP16* and C185S in *USP22*.

To generate stable cell lines that inducibly express each DUB, plasmids encoding either wild-type or catalytically-inactive DUBs were transfected into parental Flp−In T-REx 293 cells (Invitrogen) and stable cell lines were established following the manufacturers’ instructions. These cells were maintained in DMEM (Dulbecco’s Modified Eagle Medium) supplemented with 10% calf serum and 1% penicillin/streptomycin/glutamine. Doxycycline at 1 μg/mL was used to induce DUB expression 48 h before harvest.

### Affinity purification of 3XFlag-DUBs

Typically, DUB expression was induced for 48 h and cells were grown to ∼90% confluency and harvested by scraping in PBS. To obtain whole-cell extracts, cell pellets from five 15 cm dishes were resuspended in 1.5 mL cold lysis buffer (20 mM Tris, pH 7.5, 300 mM NaCl, 1 mM 4-benzenesulfonyl fluoride hydrochloride (AEBSF), 0.5% Triton X-100) and incubated on ice for 30 min. Cell lysates were centrifuged at 20,000 x *g* for 20 min and the clear supernatant was incubated with 100 µL pre-equilibrated anti-Flag M2 agarose (Sigma) for 3 h at 4 °C with rocking. After the unbound fraction was removed, the resin was washed five times with lysis buffer. Bound proteins were eluted with elution buffer (20 mM Tris, pH 7.5, 75 mM NaCl, 0.25 mM AEBSF, 0.125% Triton X-100, 1 mg/mL 3XFlag peptide) by incubation at 4°C for 30 min. Elution was repeated 3 times and the eluates were combined. Subsequently, 2 mM DTT was added to the eluates before they were concentrated (Amicon Ultra-0.5 centrifugal 3K filter device) and stored at −80°C.

### Qualitative Ub-nucleosome DUB assays

Ub-nucleosomes (50 nM) were incubated with each DUB in assay buffer (100 mM Tris, pH 7.6, 2 mM MgCl_2_, 2 mM DTT, 0.05% Brij35, 0.1 mg/mL BSA, 100 mM NaCl) in a 5 µL reaction. USP2cc was used at 5 nM. The amounts of affinity-purified DUBs were normalized based on immunoblotting for the Flag-tag. The reactions were allowed to proceed for 30 min at 37°C and then stopped by addition of 20 mM N-ethylmaleimide (NEM). The extents of deubiquitination were analyzed by 6% native-PAGE with acrylamide:bis-acrylamide ratio of 37.5:1 in a Mini-PROTEAN Tetra Cell (Bio-Rad). The gel was pre-run at 4 °C for 15 min at 130 V in 0.2X TBE (45 mM Tris-borate, 1 mM EDTA). 1.5 μL 50% (w/v) sucrose was added to each 5 µL reaction to be loaded and run for 2 h at 130 V at 4°C. The gels were stained with ethidium bromide and scanned on the GE Typhoon FLA9000 scanner. In the reactions with 3X-Flag-BAP1, NaCl was added to a final concentration of 0.3 M prior to loading on the gel in order to dissociate the enzyme from DNA.

### Quantitative Ub-nucleosome DUB assays

A fixed concentration of the DUB was added to increasing concentrations of the Ub-nucleosome substrate in a 15 µL reaction in the assay buffer (20 mM Tris, pH 7.6, 2 mM MgCl_2_, 60 mM NaCl, 2 mM DTT, 0.05% Brij35, 0.02% Triton X-100, 30 µM AEBSF and 0.4 mg/mL BSA). Fluorescently-labeled free-Ub sensor (Atto532-tUI; 250 nM) was added to each reaction to monitor the released Ub in real time. Upon addition of the DUB, fluorescence was measured continuously on a Horiba FluoroMax 4 fluorimeter at 30 °C (λ_ex_ = 530 nm, λ_em_ = 550 nm). Arbitrary fluorescence units were converted to HisUb concentration by using a standard curve constructed with HisUb at known concentrations. Initial velocities were determined using the linear part of the progress curves at each substrate concentration; *K*_M_ and *V*_max_ values were obtained by fitting the initial rates with the Michaelis-Menten equation using Prism. Each reaction was repeated twice. To determine accurately total Ub-nucleosome concentrations, 50 nM USP2cc was added to each Ub-nucleosome substrate and the amounts of free Ub released upon complete deubiquitination were measured using Atto532-tUI.

To determine the affinity of the recombinant USP22 DUBm for unmodified nucleosomes, competition enzyme assays were performed by titrating 17–4,400 nM of unmodified nucleosome into reactions containing 250 nM Atto532-tUI, 260 nM H2BKc120Ub nucleosome, and a fixed amount of recombinant USP22 DUBm. Assay conditions were as described above. The calculated initial velocities were fitted with the following equation in Prism to determine *K*_i_ for the nucleosomes (Cheng and Prusoff, 1973):

$$Y=Bottom+\frac{Top-Bottom}{1+10^{X-LogEC50}}$$

$$\mathrm{Log}\left(EC50\right)=\mathrm{Log}{\left(10\right.}^{\mathrm{Log}\left(K_{i}\right)}*\left.\left(1+\frac{\left[H2BKc120Ub\right]}{K_{d}}\right)\right)$$

X is the concentration of unmodified nucleosomes. Y is the initial rate of deubiquitination. K_d_ is the binding constant between H2BKc120Ub nucleosomes and the USP22 DUBm.

## Results

### Synthesis of site-specific ubiquitinated histones through chemical ligation

Ub and histones are relatively small proteins that can be produced in large quantities as recombinant proteins expressed in *E. coli*. Importantly, they can be refolded efficiently from their fully-denatured states. This property allows use of both Ub and histones in various chemical derivatization reactions under denaturing conditions, thereby making it possible to explore non-enzymatic strategies to generate covalent Ub-histone conjugates. Previously, Castaneda et al. (Castaneda C. et al., 2011) described a strategy to produce all-native polyubiquitin chains of defined linkages using a silver-mediated condensation reaction between the C-terminus of the donor Ub and the ε-amine of a specific lysine (henceforth referred to as the “acceptor lysine”) of a second Ub. The major challenge of this approach is to distinguish the acceptor lysine from all the other lysine residues in both the donor and acceptor Ub molecules. Castaneda et al. solved this problem by incorporation during translation in *E. coli* of an unnatural amino acid, Boc-lysine, at the position of the desired acceptor lysine. After isolation of the recombinantly-expressed protein, all the other lysine residues, as well as the N-terminal primary amine, were blocked by an orthogonal protecting group, allyloxycarbonyl (Alloc). Sequential blocking and deblocking of the Alloc or Boc protecting groups allowed specific ligation between the C-terminus of a donor Ub and the acceptor lysine.

To define chemically the desired acceptor lysine on a histone, we first attempted to incorporate a Boc-Lysine in H2B at position 120 using the same amber codon suppression system utilized by Castaneda et al. (Castaneda C. A. et al., 2011). Unfortunately, poor expression and difficulty in purifying H2BK120Boc away from contaminating DNA required an alternative strategy. Because H2A and H2B both lack native cysteine residues, we decided to mutate the desired acceptor lysine codon to encode cysteine and to then utilize reaction with ethyleneimine (also known as aziridine) to convert the single cysteine to *S*-aminoethyl-cysteine (K_C_), a close analog of a lysine. The K_C_ residue is an efficient acceptor in silver-mediated condensation and the resulting linkage is identical to that of a natural Ub–lysine isopeptide bond except for having the lysine γ-methylene group replaced by sulfur. Using H2BK120Ub as an example, the basic steps of this strategy are described in Figure 1: 1) activation of the C-terminus of 6xHis-tagged Ub by the E1 Ub-activating enzyme to generate HisUb-SR; 2) protection of available amines (N-terminus, histidine and lysine side chains) in HisUb-SR by Alloc; 3) protection of the sole cysteine sidechain in H2BK120C by reaction with methyl methanethiosulfonate (MMTS); 4) protection of available amines in H2BK120C-S-S-CH3 by Alloc; 5) deblocking the cysteine with TCEP followed by *S*-aminoethylation with ethyleneimine; 6) ligation of the two proteins by silver-mediated condensation; (7) removal of all the Alloc groups. Finally, the HisUb-H2BK_C_120 conjugate is purified by Ni-NTA affinity chromatography under denaturing conditions, refolded with the other three core histones to obtain histone octamers, and assembled into nucleosomes.

### Characterization of the reaction intermediates and products by mass spectrometry

Steps 1–5 in Figure 1 utilize well-established chemical reactions and we found that the yield at each step was nearly 100%. Reaction intermediates were characterized by Electrospray Ionization Mass Spectrometry (ESI-MS) (**I**) HisUb could be produced in large amounts in *E. coli* and purified to homogeneity by Ni-NTA affinity chromatography (∼250 mg from 6 L culture). Analysis by ESI-MS showed that purified HisUb has the expected molecular weight (Supplementary Figure S1A). (**II**) The generation of HisUbSR thiolester (SR: 2-mercaptoethane sulfonate) was confirmed by ESI-MS by the addition of 125 Da to the HisUb (Supplementary Figure S1B). The approach developed by El Oualid et al. (El Oualid et al., 2010) afforded complete thioesterification of HisUb after 6 h incubation at 37°C. The HisUbSR was then dissolved in 0.4% TFA/H_2_O to stabilize the thiolester. (**III**) Variable amounts of Alloc addition to HisUbSR were observed (Supplementary Figures S1C, D). HisUbSR has 15 potential sites for reaction with Alloc-OSu: seven lysine side chains, one N-terminal amine, and seven histidine side chains. A single Alloc adds 84 Da. The ESI-MS results showed that 10 to 15 Alloc groups were attached to HisUbSR. This variability is most likely due to partial modification of the seven histidines by Alloc, which does not appear to affect the specificity of the ligation step (see below). (**IV**) H2BK120C was expressed in *E. coli* and purified from inclusion bodies as previously described (Dyer et al., 2004) except that 1 mM acetic acid was included in the dialysis buffer in the final step to help minimize oxidation of the cysteine without the addition of β-mecaptoethanol or DTT; the molecular weight was confirmed by ESI-MS (Supplementary Figure S2A). (**V**) MALDI-TOF MS confirmed the complete protection of the cysteine by MMTS, which adds 46 Da to the molecular weight of H2BK120C (data not shown). This protection was maintained during the subsequent Alloc blocking step. (**VI**) There are 23 potential Alloc-OSu reacting groups in H2BK120C-S-S-CH3: 19 lysine side chains, one N-terminal amine, and three histidine side chains. ESI-MS showed between 21 and 23 Alloc groups were added to the protein. Again, this heterogeneity is most likely the result of partial blocking of the three histidines (Supplementary Figure S2B). (**VII**) Cysteine deprotection by treatment with TCEP results in the loss of 46 Da (Supplementary Figure S2C). Subsequent to the cysteine deprotection and alkylation by ethyleneimine, it was found that Alloc groups attached to histidine side chains were largely lost (Supplementary Figure S2D). The conversion of cysteine to *S*-aminoethylcysteine adds 43 Da and the predominant species observed by ESI-MS corresponds to H2BK_C_120 with 20 Alloc groups attached (Supplementary Figure S2D).

Unlike the several previous steps, the silver-mediated chemical ligation (Step 6) was relatively inefficient and returned yields of 10–40%. Formation of ubiquitinated histones (both H2BK_C_120Ub and H2AK_C_119Ub) was observed by SDS-PAGE (Figure 2A). To test the specificity of Ub attachment, (Alloc)H2BK120C (whose cysteine was not alkylated) was used in the negative control reaction (Figure 2A). We consistently observed a small amount of Ub-histone species generated in the negative control reaction (denoted by #). The chemical nature of the Ub attachment in this species is unclear as it is not cleaved by USP2cc, a non-specific DUB derivative that is commonly used to deubiquitinate Ub conjugates (Kim et al., 2011) (data not shown). Immunoblotting with antibodies that specifically recognize the isopeptide linkage in H2BK_C_120Ub or H2AK_C_119Ub confirmed the specificity of Ub attachment (Figure 2B). Notably, the Ub-histone species generated in the negative control reactions were not recognized by these antibodies. The bands corresponding to ubiquitinated histones were cut from the gel and sent for trypsin digestion and LC-MS/MS analysis. The spectra obtained showed a 157 Da adduct on the cysteine residue, which confirms alkylation of the cysteine by ethyleneimine (+43 Da) and addition of Gly-Gly that remained from Ub after trypsin digestion (+114 Da) (Figure 2C). The H2BK_C_120Ub and H2AK_C_119Ub proteins were refolded as 1:1 mixtures with H2A and H2B, respectively. The refolded dimers were then purified by gel filtration and the intact proteins analyzed by LC-MS (Figure 2D). The observed molecular weights confirmed that all the Alloc protecting groups had been removed efficiently.

**FIGURE 2:**
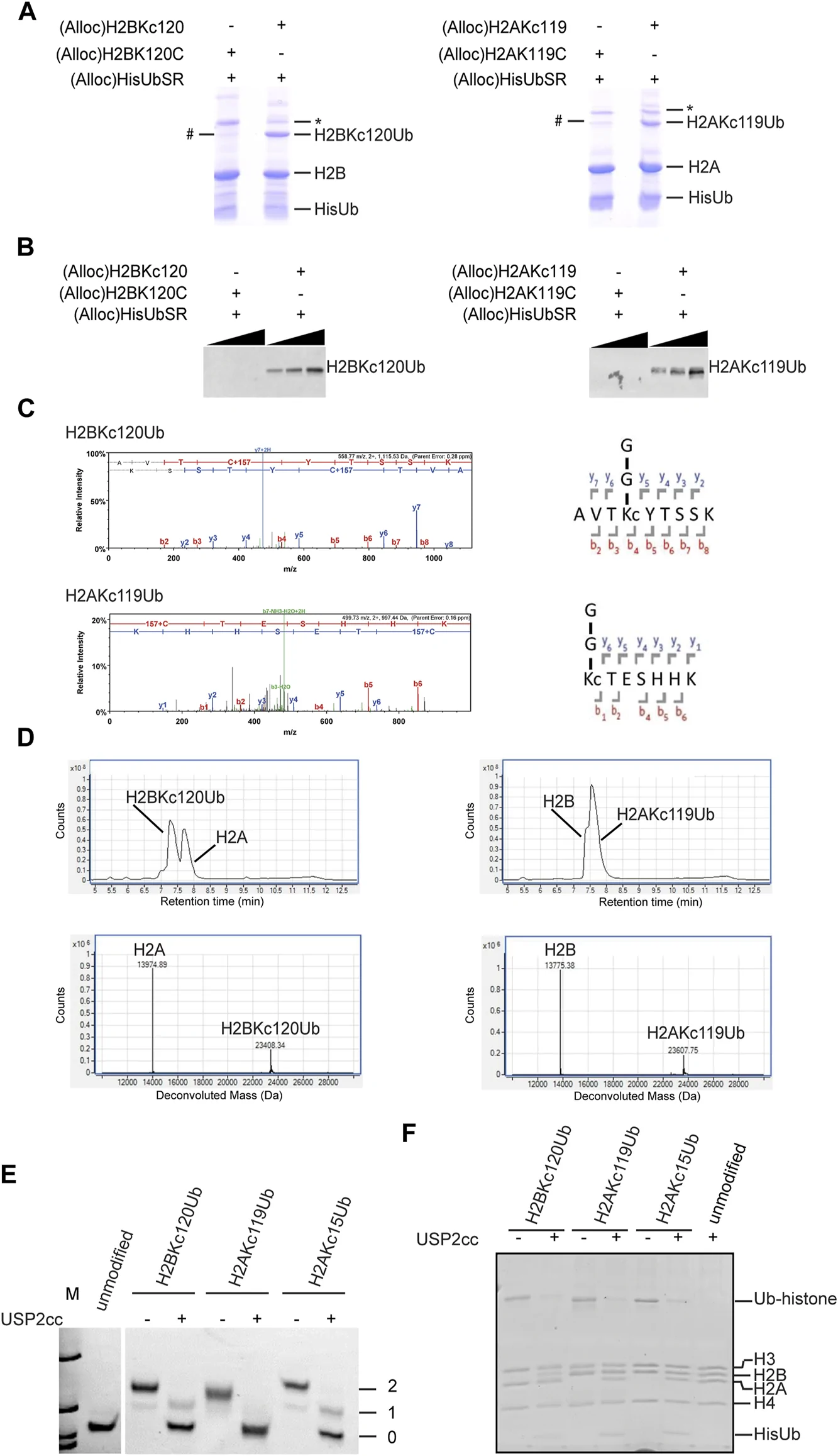
Characterization of H2BK_C_120Ub and H2AK_C_119Ub. **(A)** The chemical ligation reactions were analyzed by SDS-PAGE and Coomassie Blue stain. # denotes an unknown Ub-histone species observed in the negative control reactions with H2BK120C or H2AK119C. * is a dimeric form of H2B or H2A, possibly in aggregates. **(B)** Western blot analysis of the samples in **(A)** with linkage-specific antibodies against H2BK120Ub (left, Cell Signaling #5546)) or H2AK119Ub (right, Cell Signaling #8240). These results confirm the specificity of Ub attachment. **(C)** LC-MS/MS spectra of trypsin-digested H2BK_C_120Ub and H2AK_C_119Ub. Shown are the MS2 spectra of the tryptic peptides spanning the Ub attachment sites. The +157 Da modification on cysteine represents alkylation by ethyleneimine (+43 Da) and addition of Gly-Gly from Ub (+114 Da). **(D)** ESI-MS analysis of purified histone dimers containing Ub-histone conjugates generated by chemical ligation. Shown are the LC elution profiles (top panels) and the corresponding MS spectra (bottom panels). H2A calculated mass 13,974.2, observed 13,974.55; H2BK_C_120Ub calculated mass 23,407.8, observed 23,408.27; H2AK_C_119Ub calculated mass 23,607.1, observed 23,607.6; H2B calculated mass 13,774.9, observed 13,775.23. **(E, F)** Deubiquitination of Ub-histone conjugates generated by chemical ligation. Mononucleosomes containing H2BK_C_120Ub, H2AK_C_119Ub, or H2AK_C_15Ub were incubated with or without USP2cc and products were analyzed by native PAGE and ethidium bromide staining **(E)** or SDS-PAGE and Coomassie stain **(F)**. Numbers in **(E)** refer to the number of Ubs in the nucleosome.

### Ubiquitinated histones generated by chemical ligation are efficient substrates for DUBs

Using the method described above, we generated H2BK_C_120Ub, H2AK_C_119Ub, and H2AK_C_15Ub. Each Ub-histone was refolded with other core histones to reconstitute the histone octamers; these were then assembled into mononucleosomes with 147mer DNA of the 601 nucleosome positioning sequence by salt dilution as described (Long et al., 2014). To test if the ubiquitinated mononucleosomes generated by this method were susceptible to enzymatic deubiquitination, they were incubated with the non-specific DUB USP2cc and the products were analyzed by native-PAGE (Figure 2E) and SDS-PAGE (Figure 2F). Both analyses showed that all three ubiquitinated histones were efficiently hydrolyzed. Because addition of a single Ub could retard the nucleosome mobility, it was possible using native-PAGE to resolve nucleosomes containing one or two copies of Ub; this could be a useful approach to study potential asymmetry or cooperativity of nucleosome (de)ubiquitination.

### Specificities of selected histone DUBs

Many histone DUBs have been reported in the literature, but few have been tested to compare activities against a panel of homogeneous ubiquitinated nucleosomes *in vitro*. For this purpose, we chose four well-established human histone DUBs, BAP1, USP16, USP22 and USP3 to use with our chemically-ligated ubiquitinated nucleosomes. For each DUB, we created stable HEK293 cell lines to inducibly express the 3xFlag-tagged enzyme either as a wild-type (C) or a catalytically-inactive form whose active-site cysteine was mutated to serine (S). The DUBs and their associated factors were affinity purified on anti-Flag agarose beads and eluted under native conditions with Flag peptide (Figures 3A, B). Consistent with previous reports, each DUB was associated with other cellular proteins that co-purified (Sowa et al., 2009; Scheuermann et al., 2010; Lang et al., 2011), but currently we do not know the identities of the co-purified proteins, of which some are likely to be contaminants (Figure 3B). It is also possible that multiple DUB complexes were recovered from the one-step affinity purifications. Nonetheless, the conclusions we draw from activity assays rely on the comparison between wild-type and mutant DUBs purified in parallel and normalized to the amounts of the DUB proteins (Figure 3A). Silver-stained SDS-PAGE profiles of the eluates show strong similarities of proteins co-purified with wild-type DUBs and their mutant counterparts (Figure 3B).

**FIGURE 3:**
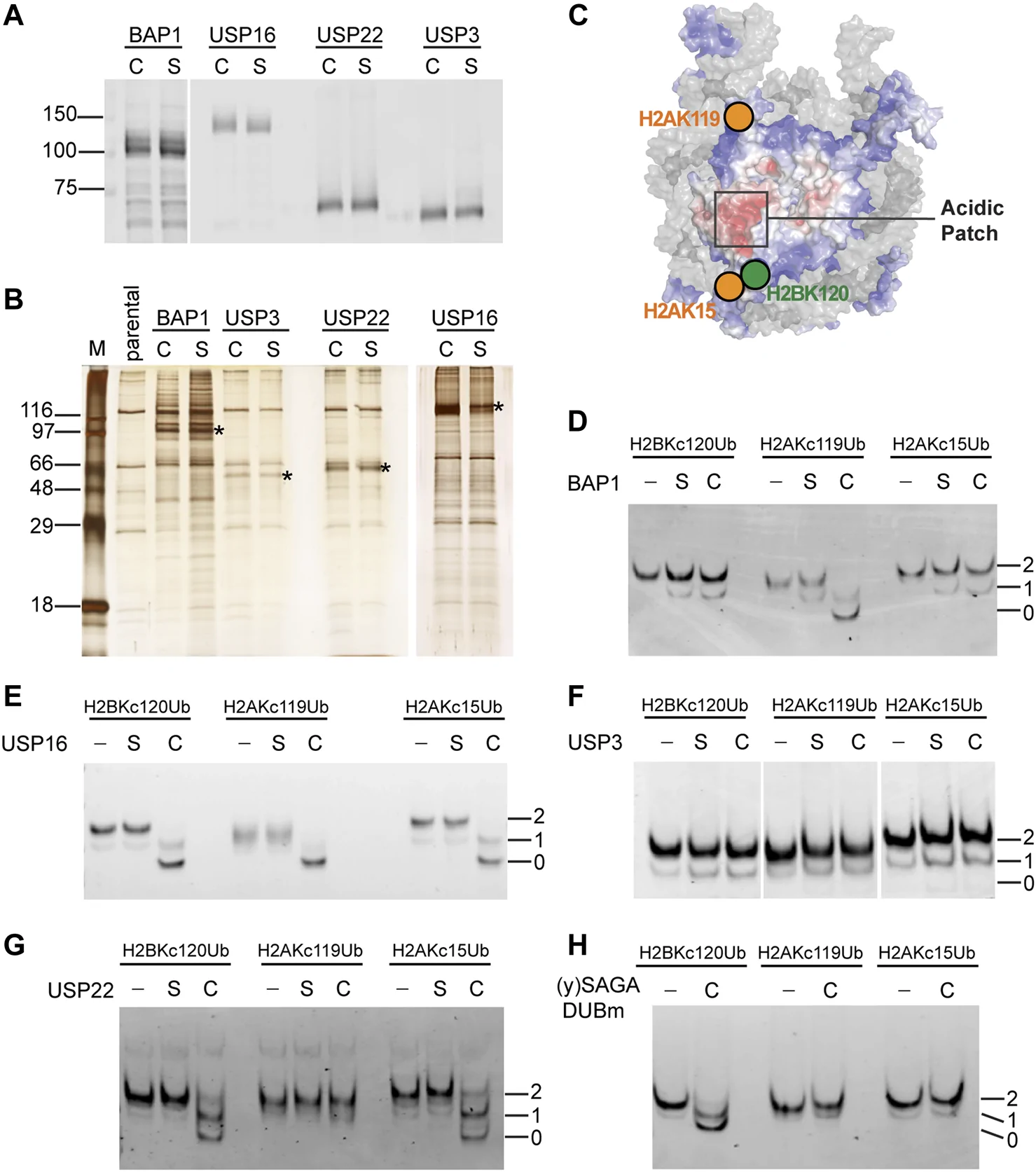
Distinct substrate specificities of affinity-purified human DUBs are revealed by use of chemically ubiquitinated nucleosomes. **(A)** The amounts of affinity-purified wild-type (*C*) and catalytically-inactive Cys-to-Ser mutant (*S*) DUBs were normalized based on immunoblots with an anti-Flag antibody. **(B)** Silver-stained gel of affinity-purified DUBs. Immunoprecipitants from parental untagged cells were loaded as a control. *Asterisks* indicate the bands that correspond to the 3xFlag-tagged DUBs. **(C)** Illustration of the spatial relationship of the tested ubiquitination sites on a nucleosome (adapted from Dos Santos Passos et al., 2021). Affinity**-**purified **(D)** BAP1, **(E)** USP16, **(F)** USP3, and **(G)** USP22 were incubated with the indicated nucleosome substrates (50 nM) at 37°C for 30 min and the reaction products were analyzed by native PAGE followed by ethidium bromide staining. *C*, *S*, and–indicate wild-type, catalytically-inactive, or no DUB added. Numbers indicate the number of ubiquitins in the nucleosome. **(H)** Recombinant yeast SAGA DUBm was expressed and purified from *E. coli.* (Samara et al., 2010). Incubation of 60 nM of the indicated nucleosome substrate with or without DUBm (40 nM) was done at 30°C for 5 min and the reaction products were analyzed by native PAGE and ethidium bromide staining.

We used Ub-aminomethylcoumarin (Ub-AMC) as a generic substrate to evaluate possible contaminating DUB activities in the affinity-purified fractions (Supplementary Figure S3). Ub-AMC hydrolysis by preparations of wild-type BAP1 or USP16 were significantly higher than with their catalytically-inactive counterparts. No appreciable Ub-AMC hydrolysis was detected with either USP3 wild-type or mutant. Both wild-type and mutant USP22 preparations showed similar low levels of Ub-AMC hydrolysis (Supplementary Figure S3C), indicating possible contamination by other DUBs.

We focused on three abundant mono-ubiquitination sites found on human histones—H2BK120, H2AK119 and H2AK15 — whose spatial localizations on the nucleosome are illustrated in Figure 3C. To assess qualitatively the substrate specificity of each DUB, we incubated the fractions with ubiquitinated mononucleosomes and monitored their activities by native PAGE (Figures 3D–G). BAP1 efficiently deubiquitinated H2AK_C_119Ub nucleosomes, but not H2BK_C_120Ub or H2AK_C_15Ub nucleosomes (Figure 3D). This is consistent with previous reports using recombinant BAP1 in a complex with its binding partner, ASXL1 (Sahtoe et al., 2016). It is likely that ASXL1 co-purified with our 3xFlag-tagged BAP1. Both the wild-type and mutant protein preparations appeared to have low levels of deubiquitinating activities against all three types of Ub-nucleosome substrates; these presumably are due to contaminating DUBs. USP16 efficiently deubiquitinated all three of the nucleosomal substrates tested (Figure 3E). We performed additional timecourse experiments and failed to detect any differences in the extent of hydrolysis among different substrates (data not shown). Thus, USP16 does not appear to differentiate H2BK_C_120Ub, H2AK_C_119Ub, and H2AK_C_15Ub nucleosomes, a result that contradicts previous reports (Joo et al., 2007) (see Discussion). To our surprise, and despite multiple attempts, the affinity-purified human USP3 had no detectable deubiquitination activity against Ub-AMC, Ub-histone, or Ub-nucleosome substrates (Figure 3F and data not shown). Finally, USP22 showed similar deubiquitination activities against the H2BK_C_120Ub and H2AK_C_15Ub nucleosomes, but little activity was observed with H2AK_C_119Ub nucleosomes (Figure 3G). The selectivity of USP22 against these different ubiquitination sites was then quantified as described below.

Although contaminating DUB activities were detected with wild-type and mutant USP22 using Ub-AMC as a substrate (Supplementary Figure S3C), we did not detect any activity against Ub-nucleosome substrates in the USP22 mutant fraction; this underscores the importance of using cognate substrates. Human USP22 is part of a DUB module (DUBm) associated with the SAGA histone acetyltransferase complex, which is conserved throughout eukaryotes (Zhang et al., 2008). H2BK120Ub is also conserved in all eukaryotes (equivalent to H2BK123Ub in yeast), whereas H2AK13/15Ub and H2AK118/119Ub have not been observed in yeast to our knowledge. Thus, we tested the specificity of the USP22 yeast homolog, yUbp8, which was produced as a recombinant protein in *E. coli* together with yeast Sgf11, Sus1, and Sgf73, which comprise yeast SAGA DUBm (Samara et al., 2010) (Figure 3H). We did not include a catalytically-inactive mutant yUbp8 control because *E. coli* do not have Ub or DUBs. Interestingly, yeast SAGA DUBm could release Ub from nucleosomes containing H2BK_C_120Ub but not H2AK_C_15Ub or H2AK_C_119Ub; thus, differences between yeast and human SAGA DUBm appear to have evolved to accommodate additional ubiquitinated nucleosomal signals.

### A quantitative DUB assay to characterize kinetic properties of USP22-family DUBs

Among the ∼80 human DUBs, USP22 is closely related to USP27X and USP51. USP22 has 82% and 70% sequence identity with USP27X and USP51, respectively. The three DUBs are interchangeable subunits of the DUBm (Atanassov et al., 2016) where they compete for binding to ATXN7L3 and ENY2; incorporation into the DUBm is required for the DUB catalytic activities. However, USP27X and USP51 do not associate with the SAGA acetyltransferase complex. Depletion of each DUB had subtle effects on global H2BK120Ub levels and affect transcription of overlapping but not identical sets of genes. Another report demonstrated that USP51 targets H2AK15Ub during DNA damage response (Wang et al., 2016). These observations raise the question of whether these highly-related DUBs have the same substrate specificities and how those specificities may instruct their respective regulatory functions *in vivo*.

To address these questions, we employed a fluorescence-based real-time DUB assay using a sensor designed to bind free, unconjugated Ub (Choi et al., 2019). The sensor, Atto532-tUI, binds to free Ub exceptionally tightly (*K*_d_ = 70 pM) and with > 10^6^ fold preference over conjugated Ub. Using this sensor, Ub released by DUBs can be captured quantitatively to form a complex that can be monitored by an ∼4-fold increase in Atto532 fluorescence (Choi and Cohen, 2023). With this assay, we determined the Michaelis-Menten kinetics of affinity-purified USP22 as well as recombinant DUBm complexes purified from insect cells that expressed USP22, USP27X or USP51, (Atanassov et al., 2016) (Figures 4A,B).

**FIGURE 4:**
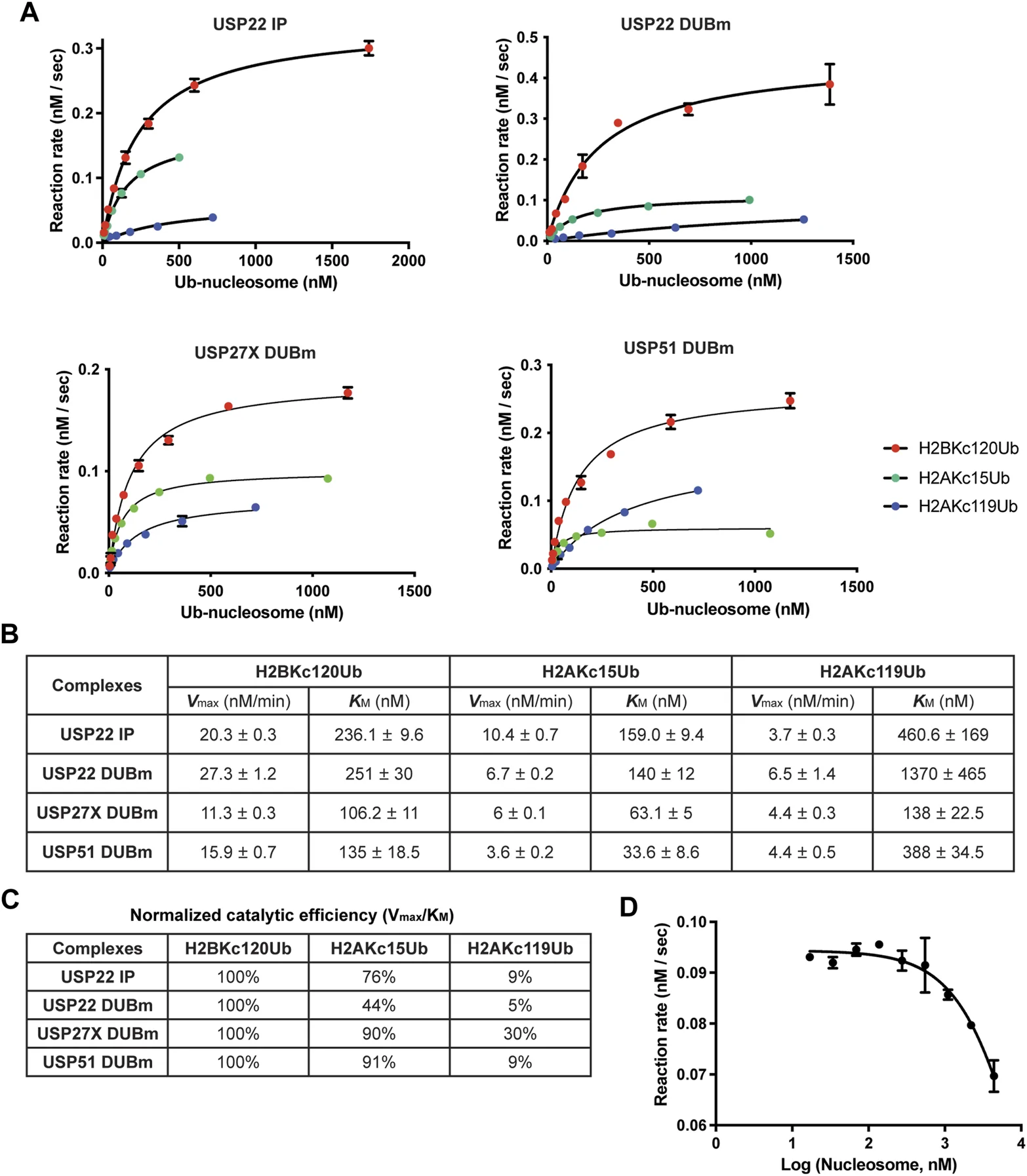
Michaelis-Menten enzyme kinetics of USP22-family DUBs. **(A)** The enzyme kinetics of 3xFlag-USP22 immuniprecipitants from mammalian cells (USP22 IP), recombinant USP22 DUBm, recombinant USP27X DUBm, and recombinant USP51 DUBm, were determined with a fluorescence-based real-time assay using Atto532-tUI, a free Ub sensor. Shown are mean values and error bars from two replicates fitted with the Michaelis-Menten equation. **(B)** *V*_max_ and *K*_M_ values and their standard errors determined from the fits shown in **(A)**. **(C)** Catalytic efficiency (*V*_max_/*K*_M_) for each nucleosome substrate is normalized to that of H2BK_C_120Ub. **(D)** Fixed concentrations of recombinant USP22 DUBm and H2BK_C_120Ub nucleosomes were incubated with increasing concentrations of unmodified nucleosomes to determine the *K*_i_ of nucleosomes. Shown are mean and S.D. values from fits to a competitive inhibition model. Data are from two replicates.

Because the concentration of *active* DUB in each preparation was not known, we could not directly compare the *V*_max_ values of the different DUBs; nonetheless, we could compare their *K*_M_ values and substrate selectivities. We determined the catalytic efficiency (*V*_max_/*K*_M_) for each substrate and normalized to that of H2BK_C_120Ub, which is the preferred substrate (Figure 4C). The results showed that all four DUBm complexes discriminate against H2AK_C_119Ub nucleosomes and that both increased *K*_M_ and decreased *V*_max_ were responsible. In contrast, all four DUBm complexes can hydrolyze H2AK_C_15Ub nucleosomes with similar efficiencies. Notably, the USP51 DUBm showed the lowest *K*_M_ (33.6 nM) for H2AK_C_15Ub nucleosomes, which suggests that this complex may be preferentially recruited to DNA damage sites where H2AK13/15Ub nucleosomes are found to cluster. Finally, by performing a competition assay with the USP22 DUBm, we determined that the *K*_i_ for unmodified nucleosomes is 6 μM (Figure 4D), which indicates that the Ub moiety contributes significantly to enzyme-substrate binding.

## Discussion

### A non-enzymatic method to generate chemically-defined site-specific ubiquitinated histones that are susceptible to enzymatic deubiquitination by DUBs

In comparison with previously published strategies that use SPPS, our method is more economical and accessible to most biochemistry laboratories. Steps 1-5 in the workflow are highly efficient, exhibiting nearly 100% yield, whereas Step 6 is the most limiting and showed yields that ranged from 10 to 40%. Because Ub and histones can be produced in *E. coli* in large quantities, those overall yields in practice permit a wide variety of downstream applications including large-scale DUB assays and structural analyses. In recent years, several key E3 ligases that catalyze formation of H2AK13/15Ub, H2AK118/119Ub or H2AK125/127129Ub have been optimized to perform efficient ubiquitination reactions *in vitro* (Mattiroli et al., 2012; McGinty et al., 2014; Uckelmann et al., 2018; Witus et al., 2021). However, it remains challenging to separate unmodified, singly-ubiquitinated, and multi-ubiquitinated nucleosomes in order to generate homogenous species; in this regard, our method offers a distinct advantage. In addition, our approach can be applied to install Ub (or, in principle, other Ub-like molecules) at any site in any type of histone without prior knowledge of the cognate E3 ligase or the need to prepare active E3 ligases for use *in vitro*.

Our synthetic approach was adapted from the method developed by Castaneda et al. to assemble polyUb chains of specific lengths and linkages (Castaneda C. et al., 2011). Several key changes were implemented in order to accommodate unique properties of histone proteins. First, a 6xHis-tag was added to the N-terminus of Ub to facilitate separation of ubiquitinated from unmodified histones after silver-mediated ligation and Alloc removal (Figure 1). In this step, we performed the Ni-NTA affinity purification in 8 M urea to maintain solubility of denatured proteins. Although unreacted HisUb co-purified with HisUb-histone conjugates, the HisUb does not interfere with refolding of histone octamers and are efficiently removed during subsequent gel filtration used to purify histone octamers. Second, to avoid low-efficiency incorporation of Boc-lysine during recombinant histone expression in *E. coli*, we opted instead to introduce a mutation into the histone gene to encode cysteine at the desired site of ubiquitination. Most histones lack natural cysteines except for H3; with H3, its C110 residue can be mutated to alanine with little effect on nucleosome structure and stability *in vitro* (Gibson et al., 2016). The sulfhydryl group of cysteine can be readily and specifically alkylated by ethyleneimine to generate *S*-aminoethylcysteine (Raftery and Cole, 1963). *S*-aminoethylcysteine is nearly isosteric with lysine and, although the sidechain amine has a pKa value ∼1.1 pH units lower than that of lysine (Hermann and Lemke, 1968), it is an effective substitute for lysine in Ub conjugation systems (Piotrowski et al., 1997; Hofmann and Pickart, 1999). It has also been used as a lysine surrogate in the chemical methylation strategy developed by the Shokat group (Simon et al., 2007). Finally, solvent compositions and Alloc removal conditions have been optimized to maintain solubility of histones (see Methods).

Since our initial work (Al-Afaleq, 2016), two additional methods to generate hydrolyzable Ub-histone conjugates have been published; as with our strategy, both make use of a cysteine mutation introduced at the site of Ub attachment (Bhat et al., 2018; Chu et al., 2019). These newly-developed approaches have the advantage of avoiding the Alloc protection/deprotection steps. The approaches by Chu et al. and by us both generate a Ub-histone conjugate that has a linkage identical to a natural isopeptide bond except that a sulfur replaces the lysine sidechain γ-methylene group. We note that, although the Ub-histone derivatives were efficiently hydrolyzed by the DUBs we tested, it remains possible that, for some DUBs, the *S*-aminoethylcysteine substitution for lysine at Ub-protein isopeptide linkages will affect activity.

### Specificities of histone DUBs require careful evaluation with defined substrates

Given that Ub attached to different sites on histones have distinct functional outcomes, it is likely that histone DUBs can distinguish these different substrates. Histone DUBs may achieve specificity either by recognizing the sequence context of the ubiquitination site or by recognizing the spatial position of the attached Ub in the context of a folded nucleosome (Figure 3C). Accessory factors may play additional roles in recruiting the DUB to the site of action. Structures of the yeast SAGA DUBm (Morgan et al., 2016) and the BAP1/ASXL1 complex (Ge et al., 2023; Thomas et al., 2023) both demonstrated that nucleosome features such as the acidic patch or DNA can be critical for enzyme binding and specificity. Historically, due to the lack of homogeneous ubiquitinated nucleosomes for use as substrates, the specificities of histone DUBs have not been systematically evaluated *in vitro*. Because antibodies specific for only a small subset of Ub-histone types (i.e., H2AK119Ub and H2BK120Ub) are commercially available, it has also been difficult to address DUB specificity *in vivo*. Additionally, *in vivo* experiments have often involved DUB overexpression, an approach that can complicate conclusions about DUB specificity and function. Uckelmann et al. were the first to systematically examine recombinant histone DUBs against nucleosomal substrates containing H2AK13/15Ub, H2AK118/119Ub and H2AK125/127/129Ub, all of which were produced using cognate E3 ligases (Uckelmann et al., 2018). Using chemically ligated substrates and DUBs in complex with accessory factors from mammalian cells, our results are highly congruent with theirs. With our chemical ligation approach, we have added H2BK120Ub to the repertoire of substrates surveyed and used them to examine the USP22 family as well as other DUBs implicated in histone deubiquitination.

Notably, USP16 does not discriminate against any of the four ubiquitinated nucleosomes tested by Uckelmann et al. or by us. This is contrary to a previous report (Joo et al., 2007) that used as substrates ubiquitinated human H2B expressed in yeast. USP16 is also known as Ubp-M, as it is required for cell cycle progression through mitosis. Our results suggest that, rather than being limited to H2AK119Ub as reported by Joo et al., USP16 most likely can remove all ubiquitination marks on histones during mitosis. Consistently, it has been reported that H2BK120Ub is largely lost during mitosis (Zhiteneva et al., 2017); whether this depends on USP16 will require further investigation. USP3 is well-established as a histone DUB that functions in DNA damage repair (Nicassio et al., 2007; Lancini et al., 2014; Sharma et al., 2014). It has been shown to localize to double-strand break sites and to counteract Ub signaling at DNA damage sites upon overexpression. However, whether USP3 distinguishes among different sites of ubiquitination on nucleosomes is unclear. Both we and Uckelmann et al. found that USP3 has little intrinsic DUB activity against the general DUB substrates Ub-AMC and Ub-Rhodamine. We also failed to detect reactivity of Ub-vinylsulfone with affinity-purified USP3 (data not shown). Furthermore, we could not detect nucleosomal deubiquitination by USP3, although low levels of activity against all three types of ubiquitinated H2A-containing nucleosomes had been reported (Uckelmann et al., 2018). These observations suggest that, as isolated, USP3 was in an inactive state that requires activation by a thus-far unknown factor missing from the *in vitro* systems.

On a nucleosome, the H2AK15 and H2BK120 Ub attachment sites are in close proximity (11 Å); this led us to investigate if the USP22-family of DUBs can distinguish nucleosomes containing H2AK15Ub from H2BK120Ub. Due to its flexible C-terminal tail, Ub has the potential to sample a large conformational space when it is attached to a substrate lysine. Previously, we have used Molecular Dynamics simulations to identify conformations adopted by Ub when it is attached to either H2AK15 or H2BK120 (Dos Santos Passos et al., 2021). Those calculations predicted that Ub attached to H2AK15 and H2BK120 have distinct but substantially overlapping conformational spaces (Figure 2D in Dos Santos Passos et al., 2021). Therefore, a DUB’s specificity could depend on its ability to access different Ub conformers when it is bound to a nucleosome. Of particular interest in this regard is our finding that, whereas yeast SAGA DUBm deubiquitinates only H2BK120Ub nucleosomes, the human DUBm complexes containing USP22, USP27X or USP51 can deubiquitinate both H2BK120Ub and H2AK15Ub nucleosomes. Like other nucleosome-binding proteins, the SAGA DUBm interacts with the nucleosome acidic patch residues H2AE64, H2BE107, and H2AE61. Using the high-resolution structure of yeast SAGA DUBm in complex with H2BK120Ub-containing nucleosome (Morgan et al., 2016), we measured the distance between nucleosome acidic patch (H2AE61, δ carbon) and the Ub hydrophobic patch (I44, β carbon) to be ∼36 Å. MD calculations showed that this distance is found in a large fraction of the H2BK120Ub conformers but is represented only sparsely in the H2AK15Ub nucleosomes. Since H2AK15Ub has only been reported in vertebrates and not in yeast, conceivably, the human DUBm enzymes might have evolved from yeast SAGA DUBm by moving the Ub-binding pocket further from the nucleosome acidic patch in order to accommodate H2AK15Ub conformers. For USP51 DUBm, which has been reported to deubiquitinated H2AK15Ub at DNA damage sites (Wang et al., 2016), its structure may be optimized to target H2AK15Ub conformers, leading to the lowest *K*_M_ among the enzyme-substrate pairs we tested.

## Data Availability

The original contributions presented in the study are included in the article/Supplementary Material; further inquiries can be directed to the corresponding author.

## References

[B1] Al-Afaleq N. O. (2016). “Generation of site-specific ubiquitinated histones through chemical ligation and characterization of histone deubiquitinases,” in Biochemistry and molecular biology (Fort Collins, CO, USA: Colorado State University).

[B2] Aquila L. Atanassov B. S. (2020). Regulation of histone ubiquitination in response to DNA double strand breaks. Cells 9, 1699. 10.3390/cells9071699 PMC7407225 32708614

[B3] Atanassov B. S. Mohan R. D. Lan X. Kuang X. Lu Y. Lin K. (2016). ATXN7L3 and ENY2 coordinate activity of multiple H2B deubiquitinases important for cellular proliferation and tumor growth. Mol. Cell 62, 558–571. 10.1016/j.molcel.2016.03.030 PMC4874879 27132940

[B4] Bhat S. Hwang Y. Gibson M. D. Morgan M. T. Taverna S. D. Zhao Y. (2018). Hydrazide mimics for protein lysine acylation to assess nucleosome Dynamics and deubiquitinase action. J. Am. Chem. Soc. 140, 9478–9485. 10.1021/jacs.8b03572 PMC6070418 29991262

[B5] Castaneda C. Liu J. Chaturvedi A. Nowicka U. Cropp T. A. Fushman D. (2011a). Nonenzymatic assembly of natural polyubiquitin chains of any linkage composition and isotopic labeling scheme. J. Am. Chem. Soc. 133, 17855–17868. 10.1021/ja207220g PMC3226840 21962295

[B6] Castaneda C. A. Liu J. Kashyap T. R. Singh R. K. Fushman D. Cropp T. A. (2011b). Controlled enzymatic synthesis of natural-linkage, defined-length polyubiquitin chains using lysines with removable protecting groups. Chem. Commun. 47, 2026–2028. 10.1039/c0cc04868b PMC3190230 21212884

[B7] Chatterjee C. McGinty R. K. Fierz B. Muir T. W. (2010). Disulfide-directed histone ubiquitylation reveals plasticity in hDot1L activation. Nat. Chem. Biol. 6, 267–269. 10.1038/nchembio.315 20208522

[B8] Chen J. J. Stermer D. Tanny J. C. (2022). Decoding histone ubiquitylation. Front. Cell Dev. Biol. 10, 968398. 10.3389/fcell.2022.968398 PMC9464978 36105353

[B9] Choi Y. S. Bollinger S. A. Prada L. F. Scavone F. Yao T. Cohen R. E. (2019). High-affinity free ubiquitin sensors for quantifying ubiquitin homeostasis and deubiquitination. Nat. methods 16, 771–777. 10.1038/s41592-019-0469-9 PMC6669086 31308549

[B10] Choi Y. S. Cohen R. E. (2023). Real-time deubiquitination assays using a free ubiquitin sensor. Methods Mol. Biol. 2591, 255–267. 10.1007/978-1-0716-2803-4_15 36350553

[B11] Chu G. C. Pan M. Li J. Liu S. Zuo C. Tong Z. B. (2019). Cysteine-aminoethylation-assisted chemical ubiquitination of recombinant histones. J. Am. Chem. Soc. 141, 3654–3663. 10.1021/jacs.8b13213 30758956

[B12] Dos Santos Passos C. Choi Y. S. Snow C. D. Yao T. Cohen R. E. (2021). Design of genetically encoded sensors to detect nucleosome ubiquitination in live cells. J. Cell Biol. 220, e201911130. 10.1083/jcb.201911130 PMC7883740 33570569

[B13] Dyer P. N. Edayathumangalam R. S. White C. L. Bao Y. Chakravarthy S. Muthurajan U. M. (2004). Reconstitution of nucleosome core particles from recombinant histones and DNA. Methods Enzymol. 375, 23–44. 10.1016/s0076-6879(03)75002-2 14870657

[B14] El Oualid F. Merkx R. Ekkebus R. Hameed D. S. Smit J. J. de Jong A. (2010). Chemical synthesis of ubiquitin, ubiquitin-based probes, and diubiquitin. Angew. Chem. Int. Ed. 49, 10149–10153. 10.1002/anie.201005995 PMC3021723 21117055

[B15] Ge W. Yu C. Li J. Yu Z. Li X. Zhang Y. (2023). Basis of the H2AK119 specificity of the Polycomb repressive deubiquitinase. Nature 616, 176–182. 10.1038/s41586-023-05841-y 36991118

[B16] Gibson M. D. Brehove M. Luo Y. North J. Poirier M. G. (2016). Methods for investigating DNA accessibility with single nucleosomes. Methods Enzymol. 581, 379–415. 10.1016/bs.mie.2016.08.014 27793287

[B17] Hermann P. Lemke K. (1968). Ionisationskonstanten und Stabilitätskonstanten der Kupfer(II)-Komplexe einiger Aminosäuren und ihrer schwefelhaltigen Analoga. Hoppe-Seyler´s Z. für Physiol. Chem. 349, 390–394. 10.1515/bchm2.1968.349.1.390 5652055

[B18] Hofmann R. M. Pickart C. M. (1999). Noncanonical MMS2-encoded ubiquitin-conjugating enzyme functions in assembly of novel polyubiquitin chains for DNA repair. Cell 96, 645–653. 10.1016/s0092-8674(00)80575-9 10089880

[B19] Joo H. Y. Zhai L. Yang C. Nie S. Erdjument-Bromage H. Tempst P. (2007). Regulation of cell cycle progression and gene expression by H2A deubiquitination. Nature 449, 1068–1072. 10.1038/nature06256 17914355

[B20] Kim W. Bennett E. J. Huttlin E. L. Guo A. Li J. Possemato A. (2011). Systematic and quantitative assessment of the ubiquitin-modified proteome. Mol. Cell 44, 325–340. 10.1016/j.molcel.2011.08.025 PMC3200427 21906983

[B21] Lancini C. van den Berk P. C. Vissers J. H. Gargiulo G. Song J. Y. Hulsman D. (2014). Tight regulation of ubiquitin-mediated DNA damage response by USP3 preserves the functional integrity of hematopoietic stem cells. J. Cell Biol. 206, 2064OIA143–1777. 10.1083/jcb.2064oia143 PMC4144738 25113974

[B22] Lang G. Bonnet J. Umlauf D. Karmodiya K. Koffler J. Stierle M. (2011). The tightly controlled deubiquitination activity of the human SAGA complex differentially modifies distinct gene regulatory elements. Mol. Cell. Biol. 31, 3734–3744. 10.1128/mcb.05231-11 PMC3165722 21746879

[B23] Long L. Furgason M. Yao T. (2014). Generation of nonhydrolyzable ubiquitin-histone mimics. Methods 70, 134–138. 10.1016/j.ymeth.2014.07.006 PMC4268123 25063569

[B24] Lowary P. T. Widom J. (1998). New DNA sequence rules for high affinity binding to histone octamer and sequence-directed nucleosome positioning. J. Mol. Biol. 276, 19–42. 10.1006/jmbi.1997.1494 9514715

[B25] Luger K. Mader A. W. Richmond R. K. Sargent D. F. Richmond T. J. (1997). Crystal structure of the nucleosome core particle at 2.8 A resolution. Nature 389, 251–260. 10.1038/38444 9305837

[B26] Mattiroli F. Penengo L. (2021). Histone ubiquitination: An integrative signaling platform in genome stability. Trends Genet. 37, 566–581. 10.1016/j.tig.2020.12.005 33485674

[B27] Mattiroli F. Vissers J. H. van Dijk W. Ikpa P. Citterio E. Vermeulen W. (2012). RNF168 ubiquitinates K13-15 on H2A/H2AX to drive DNA damage signaling. Cell 150, 1182–1195. 10.1016/j.cell.2012.08.005 22980979

[B28] McGinty R. K. Henrici R. C. Tan S. (2014). Crystal structure of the PRC1 ubiquitylation module bound to the nucleosome. Nature 514, 591–596. 10.1038/nature13890 PMC4215650 25355358

[B29] McGinty R. K. Kim J. Chatterjee C. Roeder R. G. Muir T. W. (2008). Chemically ubiquitylated histone H2B stimulates hDot1L-mediated intranucleosomal methylation. Nature 453, 812–816. 10.1038/nature06906 PMC3774535 18449190

[B30] Morgan M. Jbara M. Brik A. Wolberger C. (2019). Semisynthesis of ubiquitinated histone H2B with a native or nonhydrolyzable linkage. Methods Enzymol. 618, 1–27. 10.1016/bs.mie.2019.01.003 PMC6917205 30850047

[B31] Morgan M. T. Haj-Yahya M. Ringel A. E. Bandi P. Brik A. Wolberger C. (2016). Structural basis for histone H2B deubiquitination by the SAGA DUB module. Science 351, 725–728. 10.1126/science.aac5681 PMC4863942 26912860

[B32] Nicassio F. Corrado N. Vissers J. H. Areces L. B. Bergink S. Marteijn J. A. (2007). Human USP3 is a chromatin modifier required for S phase progression and genome stability. Curr. Biol. 17, 1972–1977. 10.1016/j.cub.2007.10.034 17980597

[B33] Owen-Hughes T. Utley R. T. Steger D. J. West J. M. John S. Cote J. (1999). Analysis of nucleosome disruption by ATP-driven chromatin remodeling complexes. Methods Mol. Biol. Clift. N.J.) 119, 319–331. 10.1385/1-59259-681-9:319 10804522

[B34] Piotrowski J. Beal R. Hoffman L. Wilkinson K. D. Cohen R. E. Pickart C. M. (1997). Inhibition of the 26 S proteasome by polyubiquitin chains synthesized to have defined lengths. J. Biol. Chem. 272, 23712–23721. 10.1074/jbc.272.38.23712 9295315

[B35] Raftery M. A. Cole R. D. (1963). Tryptic cleavage at cysteinyl peptide bonds. Biochem. biophysical Res. Commun. 10, 467–472. 10.1016/0006-291x(63)90381-4 13990433

[B36] Sahtoe D. D. van Dijk W. J. Ekkebus R. Ovaa H. Sixma T. K. (2016). BAP1/ASXL1 recruitment and activation for H2A deubiquitination. Nat. Commun. 7, 10292. 10.1038/ncomms10292 PMC4729829 26739236

[B37] Samara N. L. Datta A. B. Berndsen C. E. Zhang X. Yao T. Cohen R. E. (2010). Structural insights into the assembly and function of the SAGA deubiquitinating module. Science 328, 1025–1029. 10.1126/science.1190049 PMC4220450 20395473

[B38] Scheuermann J. C. de Ayala Alonso A. G. Oktaba K. Ly-Hartig N. McGinty R. K. Fraterman S. (2010). Histone H2A deubiquitinase activity of the Polycomb repressive complex PR-DUB. Nature 465, 243–247. 10.1038/nature08966 PMC3182123 20436459

[B39] Sharma N. Zhu Q. Wani G. He J. Wang Q. E. Wani A. A. (2014). USP3 counteracts RNF168 via deubiquitinating H2A and γH2AX at lysine 13 and 15. Cell Cycle 13, 106–114. 10.4161/cc.26814 PMC3925719 24196443

[B40] Siman P. Karthikeyan S. V. Nikolov M. Fischle W. Brik A. (2013). Convergent chemical synthesis of histone H2B protein for the site-specific ubiquitination at Lys34. Angew. Chem. 125, 8217–8221. 10.1002/ange.201303844 23794525

[B41] Simon M. D. Chu F. Racki L. R. de la Cruz C. C. Burlingame A. L. Panning B. (2007). The site-specific installation of methyl-lysine analogs into recombinant histones. Cell 128, 1003–1012. 10.1016/j.cell.2006.12.041 PMC2932701 17350582

[B42] Sowa M. E. Bennett E. J. Gygi S. P. Harper J. W. (2009). Defining the human deubiquitinating enzyme interaction landscape. Cell 138, 389–403. 10.1016/j.cell.2009.04.042 PMC2716422 19615732

[B43] Thomas J. F. Valencia-Sánchez M. I. Tamburri S. Gloor S. L. Rustichelli S. Godínez-López V. (2023). Structural basis of histone H2A lysine 119 deubiquitination by Polycomb Repressive Deubiquitinase BAP1/ASXL1. bioRxiv 2023, 529554. 10.1101/2023.02.23.529554 PMC10411902 37556531

[B44] Uckelmann M. Densham R. M. Baas R. Winterwerp H. H. K. Fish A. Sixma T. K. (2018). USP48 restrains resection by site-specific cleavage of the BRCA1 ubiquitin mark from H2A. Nat. Commun. 9, 229. 10.1038/s41467-017-02653-3 PMC5768779 29335415

[B45] Vaughan R. M. Kupai A. Rothbart S. B. (2021). Chromatin regulation through ubiquitin and ubiquitin-like histone modifications. Trends Biochem. Sci. 46, 258–269. 10.1016/j.tibs.2020.11.005 PMC7954875 33308996

[B46] Wang Z. Zhang H. Liu J. Cheruiyot A. Lee J. H. Ordog T. (2016). USP51 deubiquitylates H2AK13,15ub and regulates DNA damage response. Genes Dev. 30, 946–959. 10.1101/gad.271841.115 PMC4840300 27083998

[B47] Weake V. M. Workman J. L. (2008). Histone ubiquitination: Triggering gene activity. Mol. Cell 29, 653–663. 10.1016/j.molcel.2008.02.014 18374642

[B48] Wessel D. Flugge U. I. (1984). A method for the quantitative recovery of protein in dilute solution in the presence of detergents and lipids. Anal. Biochem. 138, 141–143. 10.1016/0003-2697(84)90782-6 6731838

[B49] Witus S. R. Burrell A. L. Farrell D. P. Kang J. Wang M. Hansen J. M. (2021). BRCA1/BARD1 site-specific ubiquitylation of nucleosomal H2A is directed by BARD1. Nat. Struct. Mol. Biol. 28, 268–277. 10.1038/s41594-020-00556-4 PMC8007219 33589814

[B50] Yung-Chi C. Prusoff W. H. (1973). Relationship between the inhibition constant (KI) and the concentration of inhibitor which causes 50 per cent inhibition (I50) of an enzymatic reaction. Biochem. Pharmacol. 22, 3099–3108. 10.1016/0006-2952(73)90196-2 4202581

[B51] Zhang X. Y. Varthi M. Sykes S. M. Phillips C. Warzecha C. Zhu W. (2008). The putative cancer stem cell marker USP22 is a subunit of the human SAGA complex required for activated transcription and cell-cycle progression. Mol. Cell 29, 102–111. 10.1016/j.molcel.2007.12.015 PMC2254522 18206973

[B52] Zhiteneva A. Bonfiglio J. J. Makarov A. Colby T. Vagnarelli P. Schirmer E. C. (2017). Mitotic post-translational modifications of histones promote chromatin compaction *in vitro* . Open Biol. 7, 170076. 10.1098/rsob.170076 PMC5627050 28903997

