# Supplementary material for: Generation of site-specific ubiquitinated histones through chemical ligation to probe the specificities of histone deubiquitinases
Source: Front Epigenet Epigenom. 2023 Jul 31;1:1238154. doi: 10.3389/freae.2023.1238154 (PMC11952697; doi:10.3389/freae.2023.1238154)
Supplement: Supplementary file 1 [file DataSheet1.PDF]

Supplementary Material

Generation Of Site-Specific Ubiquitinated Histones Through Chemical Ligation To Probe the Specificities of Histone Deubiquitinases

Nouf Omar Al-Afaleq[1], Yun-Seok Choi[1], Boyko S. Atanassov[2], Robert E. Cohen[1], Tingting Yao[1*]

[1]Department of Biochemistry and Molecular Biology, Colorado State University, CO, USA

[2] Department of Pharmacology and Therapeutics, Roswell Park Comprehensive Cancer Center, Elm & Carlton Streets, Buffalo, NY

*** Correspondence:**
Tingting Yao (tingting.yao@colostate.edu)

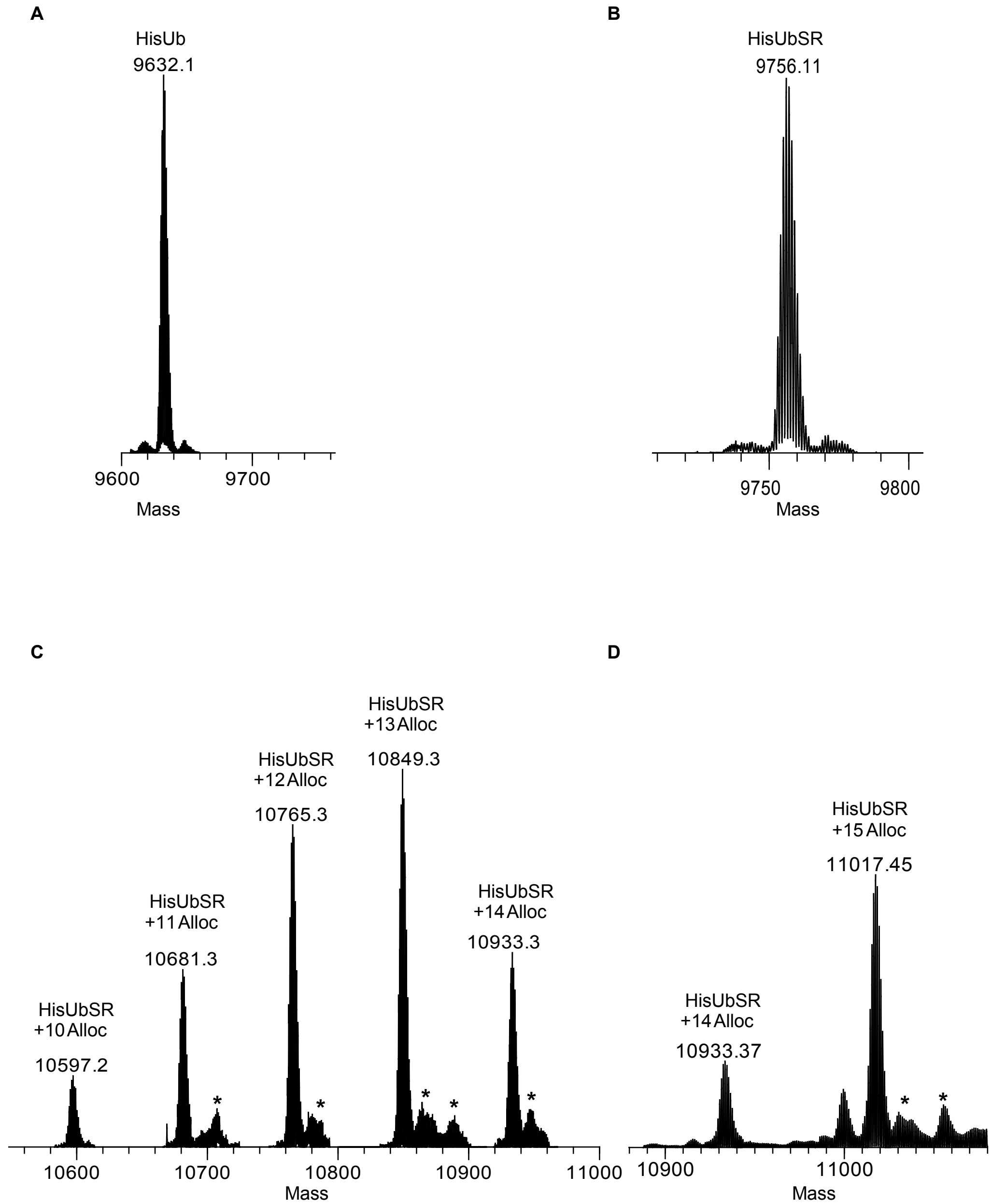

Supplementary Figure 1. ESI spectra of Ub reaction intermediates, I, II, III. (A) HisUb, calculated mass: 9632.9, observed: 9632.1; **(B)** HisUbSR, calculated mass: 9757.9, observed: 9756.1. **(C and D)** Up to 15 Alloc groups were added to HisUbSR during blocking. Each Alloc group adds 84 Da. The imidazole groups of the seven histidines can react with Alloc, but often not efficiently. Asterisks indicate possible water adducts (+18 Da), which have been noted previously in Alloc-modified proteins (Castaneda et al., 2011a).

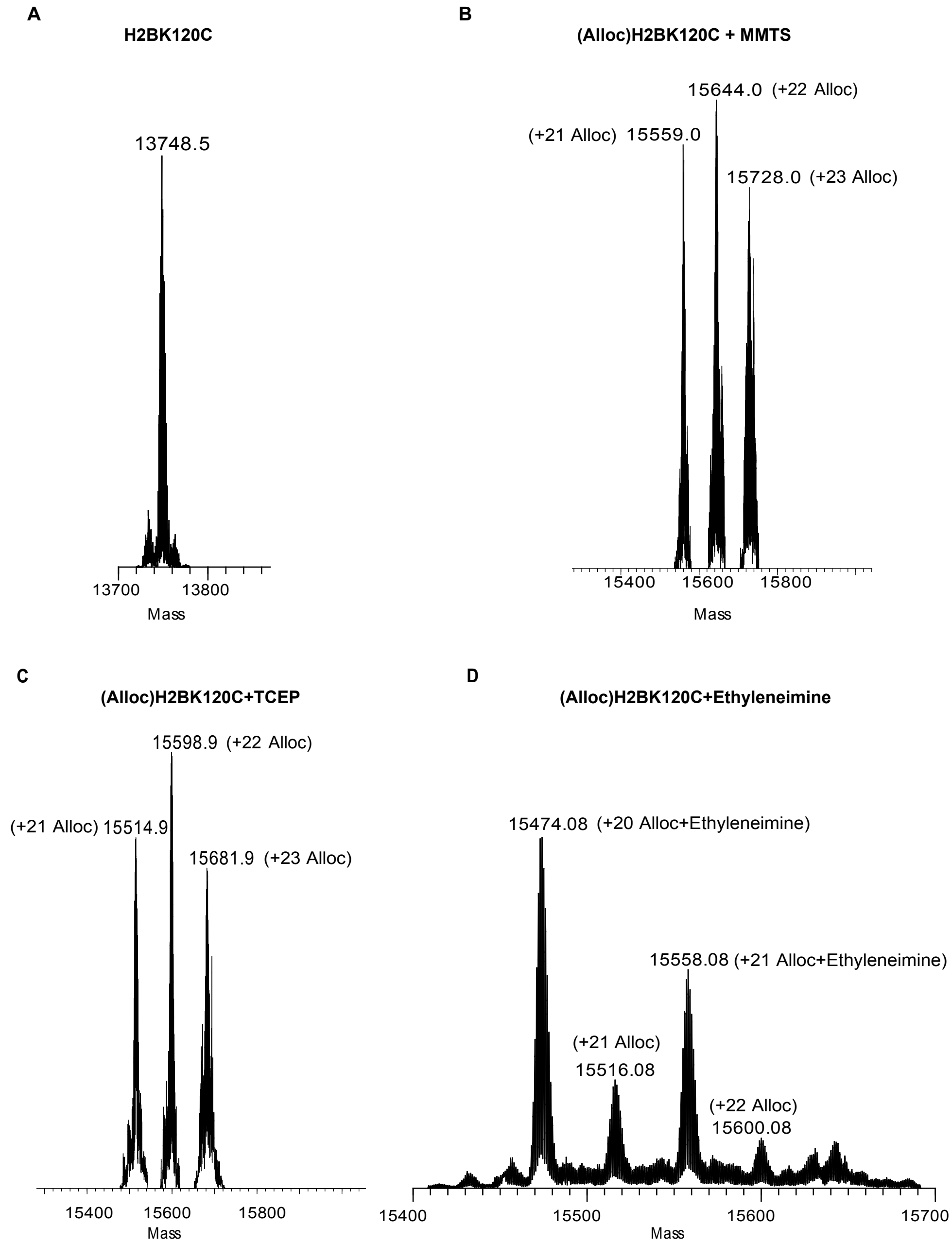

Supplementary Figure 2. ESI spectra of H2B reaction intermediates, IV, V, VI, VII. (A) Purified H2BK120C, calculated mass: 13749.9, observed: 13748.5. **(B)** H2BK120C after treatment with MMTS and *N*-(Allyloxycarbonyloxy)succinimide, Each Alloc group adds 84 Da. **(C)** Cysteine de-protection by TCEP results in the loss of 46 Da from all three species. **(D)** Cysteine alkylation by ethyleneimine adds 43 Da. The main species corresponds to the presence of 20 stable Alloc groups. Three Alloc groups, most likely attached to histidines, were partially lost during this reaction. Calculated mass: 15472.9, observed: 15474.08.

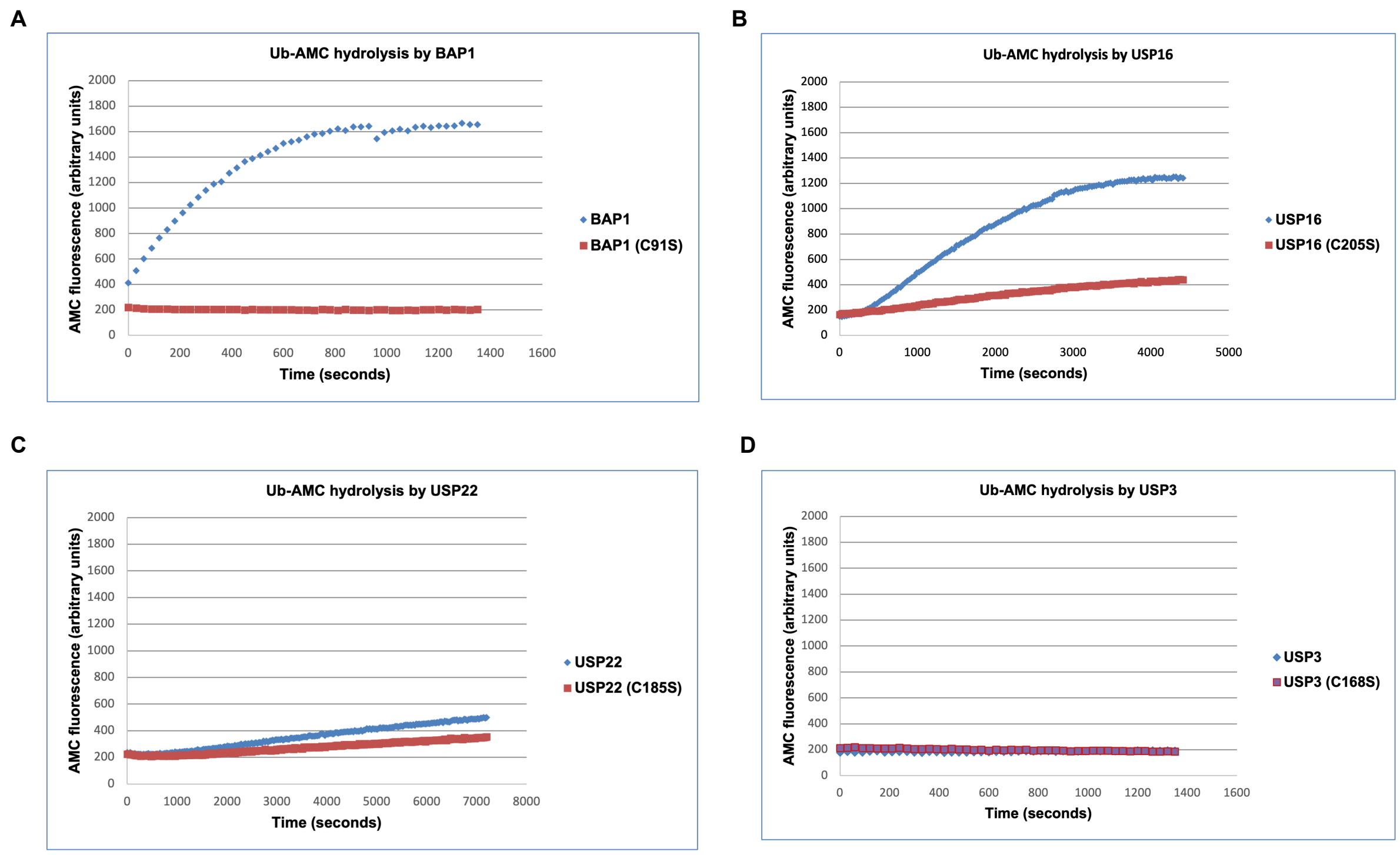

Supplementary Figure 3. Ub-AMC hydrolysis by affinity-purified DUBs. Ub-AMC (0.5 µM) hydrolysis by affinity-purified wild-type (C) or catalytically-inactive mutant (S) versions of **(A)** BAP1, **(B)** USP16, **(C)** USP22, and (**D**) USP3 was monitored continuously at 30°C on a Synergy 4 plate reader (Biotek, λex =340 nm and λem = 440 nm). Ub-AMC hydrolysis by additions of the mutant versions of the DUBs indicate possible DUB contaminants.
